# Sequential Versus Non-Sequential Polymyxin B Hemoperfusion in Severe Sepsis and Septic Shock: A Real-World Cohort Analysis of Survival in an Asian ICU

**DOI:** 10.3390/diagnostics16010173

**Published:** 2026-01-05

**Authors:** Wei-Hung Chang, Ting-Yu Hu, Li-Kuo Kuo

**Affiliations:** 1 Department of Critical Care Medicine, MacKay Memorial Hospital, Taipei 10449, Taiwan; 2 Department of Medicine, Mackay Medical College, New Taipei City 25245, Taiwan

**Keywords:** polymyxin B hemoperfusion, sepsis, septic shock, sequential therapy, survival, intensive care unit, vasoactive-inotropic score

## Abstract

**Background:** Severe sepsis and septic shock remain major causes of ICU mortality despite advances in critical care. Polymyxin B hemoperfusion (PMX-HP) is widely used in Asia for refractory endotoxemia, yet the optimal session strategy remains unclear. **Methods:** We retrospectively analyzed adult ICU patients with severe sepsis or septic shock treated with PMX-HP between 2013 and 2019 in a tertiary center in Taiwan. Patients were divided into sequential (≥2 sessions within 24 h) and non-sequential groups. The primary outcome was 28-day mortality; secondary outcomes included ICU and hospital mortality, length of stay, organ support, and vasoactive-inotropic score (VIS) changes. **Results:** Among 64 patients, 33 (51.6%) received sequential therapy. The 28-day mortality was 46.9%, with no difference between groups after adjustment for baseline severity. Patients receiving sequential PMX-HP had longer hospital stays and more frequent CRRT use, likely reflecting greater underlying disease severity rather than a causal effect of treatment sequencing. **Conclusions:** Multivariate analysis identified higher APACHE II score, positive VIS change, and CRRT requirement as independent predictors of mortality. Sequential therapy itself was not associated with improved outcomes. Prognosis in PMX-HP-treated patients is determined mainly by underlying severity and hemodynamic instability, underscoring the need for patient selection and biomarker-guided strategies rather than routine sequential use.

## 1. Introduction

Sepsis is a life-threatening syndrome caused by a dysregulated host response to infection and remains one of the most pressing global health problems. According to the Global Burden of Disease Study, sepsis affected nearly 49 million people in 2017 and contributed to more than 11 million deaths worldwide, accounting for almost one in five deaths [1]. Even in high-resource settings, the mortality rate of septic shock often exceeds 40% despite advances in antimicrobial therapy, organ support, and standardized management bundles [2,3]. The substantial burden of disease underscores the urgent need for novel adjunctive therapies to improve outcomes in critically ill patients.

A key pathophysiological driver of sepsis, particularly in Gram-negative infections, is endotoxin (lipopolysaccharide, LPS). By activating toll-like receptor 4 signaling, endotoxin triggers a cascade of pro-inflammatory cytokines, endothelial injury, vasoplegia, and microcirculatory failure that collectively lead to multi-organ dysfunction and death [4]. Importantly, endotoxemia has also been documented in non-Gram-negative infections, including viral and fungal sepsis, through mechanisms such as bacterial translocation across a compromised intestinal barrier [5,6].

Polymyxin B hemoperfusion (PMX-HP) is an extracorporeal therapy developed to selectively adsorb circulating endotoxin by binding it to polymyxin B-immobilized polystyrene fibers [7]. Unlike systemic polymyxin B administration, which is limited by nephrotoxicity and neurotoxicity, hemoperfusion confines the drug to the extracorporeal circuit and minimizes systemic exposure [7]. Early studies from Japan and Italy suggested that PMX-HP could improve hemodynamics, reduce vasopressor requirements, and potentially improve survival in patients with septic shock [8].

However, the effectiveness of PMX-HP has been questioned by subsequent large randomized controlled trials. The EUPHAS trial (2009) reported improved survival and organ function with PMX-HP in abdominal septic shock [9], but the ABDOMIX trial (2015) failed to show mortality benefit [10]. The EUPHRATES trial (2018), which targeted patients with high endotoxin activity, also did not demonstrate an overall survival advantage, although exploratory analyses suggested a possible benefit in patients with intermediate endotoxin activity levels [11]. These conflicting findings have fueled ongoing debate, and meta-analyses remain inconclusive—with some reporting mortality reductions in selected subgroups, and others finding no significant effect [12,13,14].

Geographic and epidemiological differences may partly explain these discrepancies. In East Asia, Gram-negative bacteria such as *Escherichia coli* and *Klebsiella pneumoniae* predominate as causes of sepsis, whereas Western cohorts often feature more Gram-positive or mixed infections [15]. Consequently, PMX-HP has been widely adopted in Japan and Taiwan, supported by reimbursement policies under their respective national health systems [16,17]. Yet unlike Japan, where biomarker-guided initiation using endotoxin activity assay (EAA) is common, Taiwan’s National Health Insurance covers PMX-HP without stringent criteria. As a result, initiation, frequency, and sequencing of therapy vary considerably in practice, raising questions about optimal protocols and cost-effectiveness.

One of the most controversial aspects of PMX-HP use concerns whether sessions should be administered sequentially—typically two sessions within 24 h—or non-sequentially, as a single isolated session. Proponents of sequential therapy argue that sustained or biphasic endotoxin release warrants repeated adsorption for effective clearance [18]. Early registry data from Japan suggested improved hemodynamic stability with sequential treatment [19]. However, other observational studies, including those from Taiwanese ICUs, have not found survival advantages with sequential use [20]. Moreover, sequential therapy doubles resource utilization, increases costs, and may expose patients to additional risks such as thrombocytopenia, circuit clotting, and procedure-related hypotension [21]. Methodological issues further complicate interpretation: patients must survive long enough to receive multiple sessions, creating potential immortal time bias that can exaggerate perceived benefits.

Recent studies indicate that prognosis in PMX-HP-treated patients is more strongly determined by baseline severity and organ dysfunction than by the number of sessions performed. The vasoactive-inotropic score (VIS) has emerged as a robust predictor of mortality in septic shock, with persistent or rising VIS after PMX-HP independently associated with poor outcomes [22]. Similarly, higher APACHE II scores and early requirement for continuous renal replacement therapy (CRRT) are consistent predictors of poor prognosis, irrespective of treatment sequencing [23,24]. These findings suggest that dynamic hemodynamic response and organ support requirements may be more clinically meaningful prognostic markers than sequential therapy itself.

Despite these insights, no high-quality real-world studies from Taiwan have systematically compared sequential versus non-sequential PMX-HP in terms of survival and healthcare resource utilization. Most prior work has either pooled patients regardless of sequencing or lacked detailed session-level data.

To address this gap, we conducted a retrospective cohort study of adult patients with severe sepsis or septic shock treated with PMX-HP in a Taiwanese tertiary ICU. Patients were stratified into sequential and non-sequential groups, and outcomes were analyzed with respect to 28-day mortality, length of stay, organ support, and VIS dynamics. We hypothesized that sequential therapy would not significantly improve survival compared with non-sequential therapy, and that patient prognosis would instead be driven by baseline severity and hemodynamic response. Our findings provide real-world evidence to inform patient selection, optimize clinical practice, and guide policy discussions on the cost-effective use of PMX-HP in modern sepsis care.

Rather than aiming to demonstrate a novel mortality benefit of sequential PMX-HP, the present study was designed to provide real-world validation of previously reported findings and to clarify the prognostic role of treatment sequencing and hemodynamic response in a contemporary ICU cohort.

## 2. Materials and Methods

### 2.1. Study Design and Setting

This retrospective cohort study was conducted in the medical intensive care unit (ICU) of MacKay Memorial Hospital, a 25-bed tertiary referral center in Taipei, Taiwan. The ICU admits critically ill adult patients requiring advanced monitoring and organ support, including mechanical ventilation, continuous renal replacement therapy (CRRT), and extracorporeal membrane oxygenation (ECMO). Polymyxin B hemoperfusion (PMX-HP) has been reimbursed by the Taiwan National Health Insurance (NHI) program since 2013 for patients with refractory septic shock, enabling standardized use across the study period. The study period spanned from 1 July 2013 to 31 December 2019.

### 2.2. Patient Population

All adult patients (≥20 years old) admitted to the ICU with a diagnosis of severe sepsis or septic shock were screened. Sepsis definitions were based on international guidelines in effect during the study period: prior to 2016, “severe sepsis” was defined as sepsis with organ dysfunction, and “septic shock” as persistent hypotension despite adequate fluid resuscitation; after 2016, the Sepsis-3 criteria were adopted, defining septic shock as vasopressor requirement to maintain mean arterial pressure ≥65 mmHg with serum lactate >2 mmol/L despite adequate resuscitation.

Inclusion criteria:

- Diagnosis of severe sepsis or septic shock.
- At least one session of PMX-HP during ICU admission.

Exclusion criteria:

- Age <20 years.
- Pregnancy.
- Anticipated survival <30 days due to terminal malignancy or end-stage organ disease.
- Cardiopulmonary resuscitation within 4 weeks before ICU admission.
- Do-not-resuscitate (DNR) orders or palliative care designation at admission.
- Solid-organ transplantation within 1 year.
- Known HIV infection or hemophilia.
- Allergy to polymyxin B, heparin, or extracorporeal circuit materials.
- Receipt of extracorporeal blood purification (CRRT, hemofiltration, or plasma exchange) within 24 h prior to enrollment.

### 2.3. Definitions of Sequential Versus Non-Sequential Therapy

Patients were stratified into two treatment groups:

- Sequential PMX-HP: two or more sessions administered within a 24 h interval.
- Non-sequential PMX-HP: one session only, or multiple sessions separated by >24 h.

The decision to initiate PMX-HP and determine the number and timing of sessions was made by the attending intensivist, based on clinical judgment, hemodynamic status, and response to prior therapy. Among patients classified as non-sequential PMX-HP, 7 patients received a second session more than 24 h after the first session, and no patient underwent a third PMX-HP session during the same ICU admission.

### 2.4. PMX-HP Intervention

PMX-HP was performed using a Toraymyxin^®^ cartridge (Toray Industries, Tokyo, Japan). Each session was typically prescribed for 2–6 h with a blood flow rate of 80–120 mL/min, anticoagulated with unfractionated heparin per institutional protocol. Vascular access was achieved using a double-lumen catheter inserted into a central vein. Indications included refractory septic shock with vasopressor requirement despite adequate volume resuscitation and broad-spectrum antibiotic therapy. The decision for sequential therapy was individualized based on clinical response after the first session.

### 2.5. Data Collection

Clinical, demographic, and treatment data were retrospectively extracted from electronic medical records using a standardized data collection form. Variables included:

- Demographics: age, sex, body weight.
- Comorbidities: hypertension, diabetes mellitus, chronic kidney disease, chronic liver disease, malignancy, immunosuppression.
- Severity scores: APACHE II score at ICU admission (Knaus et al., Crit Care Med 1985 [25]); SOFA score when available.
- Source of sepsis: pneumonia, intra-abdominal infection, urinary tract infection, skin/soft tissue infection, liver abscess, bacteremia, or others.
- Microbiology: pathogens identified from cultures, including multidrug-resistant (MDR) organisms.
- PMX-HP treatment details: number of sessions, session duration, time interval between sessions, sequential vs. non-sequential classification.
- Organ support: timing and use of CRRT and ECMO.
- Hemodynamic data: vasoactive-inotropic score (VIS) (Gaies et al., Pediatr Crit Care Med 2010; Koponen et al., Br J Anaesth 2019 [23,24]) measured before PMX-HP (T1), immediately after the final PMX-HP session (T2), and 24 h post-final session (T3).
- Outcomes: 28-day mortality, ICU mortality, hospital mortality, ICU length of stay (LOS), hospital LOS, and organ support requirements.

### 2.6. Outcome Measures

The primary outcome was 28-day all-cause mortality after PMX-HP initiation. Secondary outcomes included:

- ICU mortality.
- Hospital mortality.
- ICU and hospital length of stay.
- Requirement and timing of CRRT and ECMO.
- Dynamic changes in VIS (ΔVIS defined as T3–T1).
- Ventilator-free days (if recorded).

### 2.7. Statistical Analysis

Continuous variables were expressed as mean ± standard deviation (SD) or median (interquartile range, IQR), depending on distribution. Categorical variables were summarized as counts and percentages. Group comparisons (sequential vs. non-sequential) were performed using Student’s *t*-test or Mann–Whitney *U* test for continuous variables, and the chi-square or Fisher’s exact test for categorical variables.

Survival curves were estimated using the Kaplan–Meier method and compared using the log-rank test. Multivariate logistic regression was applied to identify independent predictors of 28-day mortality, adjusting for clinically relevant variables including age, APACHE II score, VIS dynamics, and CRRT requirement. Odds ratios (ORs) with 95% confidence intervals (CIs) were reported.

Subgroup analyses were pre-specified to explore outcomes according to infection source (Gram-negative vs. non-Gram-negative), presence of MDR organisms, and baseline APACHE II tertiles. Sensitivity analyses were performed by excluding patients with extreme outliers in LOS to ensure robustness of results.

All analyses were performed using IBM SPSS Statistics version 26.0 (IBM Corp., Armonk, NY, USA) and R version 4.2. Two-tailed *p* < 0.05 was considered statistically significant.

### 2.8. Missing Data Handling

Missing values for laboratory or hemodynamic data were handled by pairwise deletion, consistent with prior ICU sepsis cohort methodology. No imputation was performed to avoid introducing bias in dynamic VIS trends or outcome analyses.

### 2.9. Ethical Considerations

The study was approved by the Institutional Review Board of MacKay Memorial Hospital (IRB approval code: 18MMHIS198e, approval date: 4 March 2018). Given the retrospective and anonymized nature of the analysis, the requirement for informed consent was waived. The study adhered to the principles of the Declaration of Helsinki and institutional guidelines for research involving human subjects.

### 2.10. Data and Materials Availability

The datasets generated and analyzed in this study are not publicly available due to patient confidentiality but are available from the corresponding author upon reasonable request. Analytical code and statistical scripts used in this study are available on request. If required by the editorial office, data will be deposited in a publicly accessible repository, and accession numbers will be provided prior to publication.

### 2.11. Bias Control and Sensitivity Analyses

Given the retrospective design, potential sources of bias were carefully evaluated. Selection bias was minimized by screening all consecutive ICU admissions meeting sepsis criteria during the study period. To address immortal time bias—where patients must survive long enough to receive a second PMX-HP session—we performed sensitivity analyses excluding those who died within 24 h after the first PMX-HP treatment. Confounding by indication was mitigated through multivariate logistic regression including clinically relevant variables (age, sex, APACHE II score, VIS change, and CRRT use). Model calibration was assessed using the Hosmer–Lemeshow goodness-of-fit test, and collinearity was examined through variance inflation factors (<2.0 for all covariates). All statistical assumptions were verified before analysis. To test robustness, alternative models using Firth logistic regression yielded similar estimates for the main predictors of mortality.

## 3. Results

### 3.1. Patient Flow

**Figure 1:**
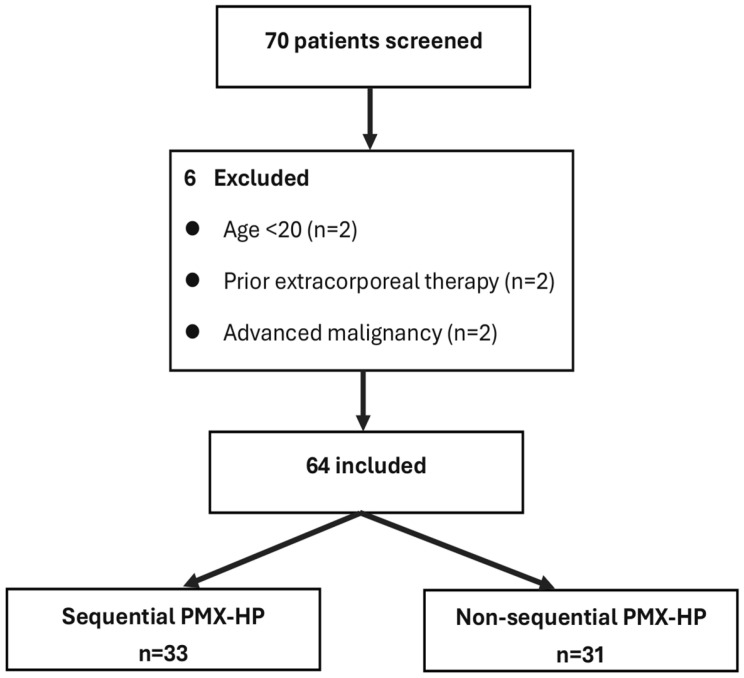
Patient enrollment flow diagram. Seventy patients with severe sepsis or septic shock were screened between 2013 and 2019. Six were excluded (age < 20 years, *n* = 2; extracorporeal therapy within 24 h prior to ICU admission, *n* = 2; advanced malignancy, *n* = 2). Sixty-four were included and stratified into sequential PMX-HP (≥2 sessions within 24 h; *n* = 33) and non-sequential PMX-HP (*n* = 31). Among them, 33 patients (51.6%) underwent sequential PMX-HP (≥2 sessions within 24 h), and 31 patients (48.4%) received non-sequential PMX-HP (one session or multiple sessions >24 h apart). Within the non-sequential group, 7 patients received a second PMX-HP session at an interval greater than 24 h, and no patient received three or more sessions in total.

A total of 70 patients with severe sepsis or septic shock were screened during the study period. Six patients were excluded (age <20 years, *n* = 2; extracorporeal therapy within 24 h prior to ICU admission, *n* = 2; anticipated survival <30 days due to malignancy, *n* = 2). Sixty-four patients were included in the final analysis (Figure 1). In the non-sequential group, 7 of 31 patients underwent a second PMX-HP session more than 24 h after the first, and none received three or more sessions.

### 3.2. Baseline Characteristics

Baseline characteristics are shown in Table 1. The mean age was 66.1 ± 12.3 years, and 67.2% were male. The median APACHE II score at ICU admission was 26 (IQR 21–32).

The leading sources of sepsis were pneumonia (29.7%), intra-abdominal infection (18.8%), and urinary tract infection (17.2%). Gram-negative organisms predominated, with *Escherichia coli* (31.3%) and *Klebsiella pneumoniae* (21.9%) most common. Multidrug-resistant (MDR) pathogens were present in 26.6%.

No significant differences were observed between sequential and non-sequential groups in age, sex, comorbidities, APACHE II score, infection source, or pathogen profile (*p* > 0.1 for all).

### 3.3. Clinical Outcomes

#### 3.3.1. Mortality

Overall 28-day mortality was 46.9% (30/64). ICU and hospital mortality were both 53.1% (34/64).

By treatment group, 28-day mortality was 45.5% in sequential vs. 48.4% in non-sequential (*p* = 0.82). ICU mortality (51.5% vs. 54.8%, *p* = 0.77) and hospital mortality (51.5% vs. 54.8%, *p* = 0.77) were also similar (Table 2).

#### 3.3.2. Length of Stay and Resource Use

Median ICU length of stay (LOS) was 9.3 days (IQR 4.4–21.1), and hospital LOS was 20.5 days (IQR 8.0–34.6). Sequential therapy was associated with longer hospital LOS (median 24.8 vs. 14.2 days, *p* = 0.03). ICU LOS showed a trend toward longer stay with sequential therapy but was not statistically significant (11.1 vs. 7.8 days, *p* = 0.09).

CRRT was required more often in the sequential group (69.7% vs. 54.8%, *p* = 0.04). ECMO was used in 3 patients (4.7%), evenly distributed.

### 3.4. Survival Analysis

**Figure 2:**
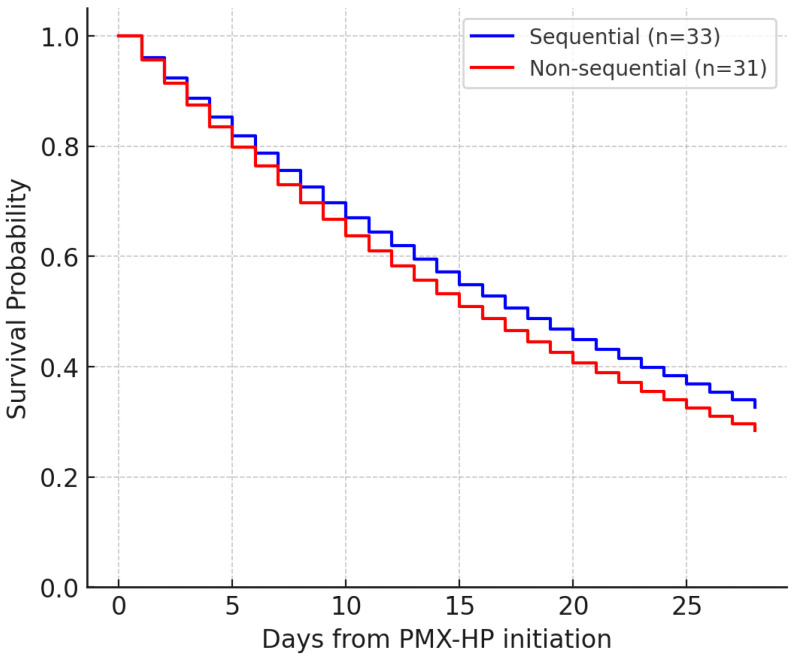
Kaplan–Meier 28-day survival curves comparing sequential and non-sequential PMX-HP groups (log-rank *p* = 0.74).

Kaplan–Meier survival analysis showed no significant difference in 28-day survival between groups (log-rank *p* = 0.74) (Figure 2). Median survival time was similar in both sequential and non-sequential cohorts.

### 3.5. VIS Dynamics and Hemodynamic Response

#### 3.5.1. Overall Trends

VIS was assessed at three time points: before PMX-HP (T1), after the last session (T2), and 24 h later (T3). At baseline (T1), the median VIS was 0.0 (IQR 0.0–0.0).

At T3, survivors had significantly lower VIS than non-survivors (median 4.5 vs. 27.8, *p* = 0.001).

#### 3.5.2. Group Comparison

**Figure 3:**
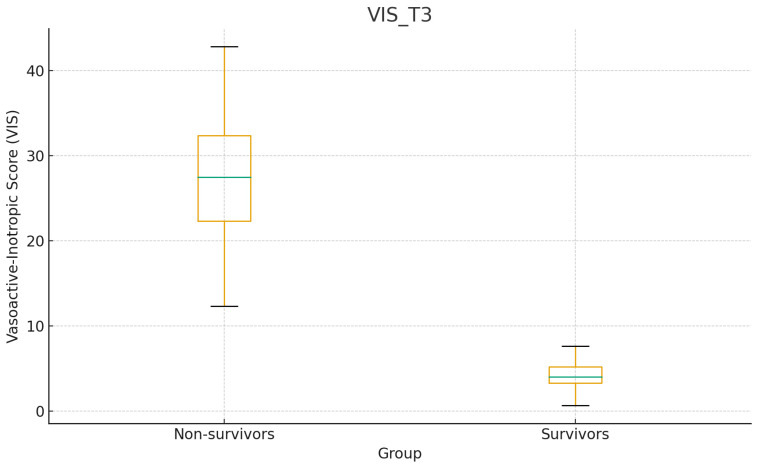
Vasoactive-inotropic score (VIS) at 24 h after the final PMX-HP session (T3) in survivors vs. non-survivors (median 4.5 vs. 27.8; *p* = 0.001). The blue horizontal line in the boxplot represents the median value of the vasoactive-inotropic score (VIS).

VIS reduction was observed in survivors across both sequential and non-sequential groups, whereas persistent elevation was common in non-survivors. No significant interaction between sequencing and VIS change was detected (*p* = 0.89) (Figure 3).

### 3.6. Subgroup Analyses

#### 3.6.1. Gram-Negative vs. Non-Gram-Negative Infections

Mortality was higher among patients with Gram-negative infections (50.0%) compared to non-Gram-negative infections (41.2%), but sequencing did not affect outcomes within either subgroup (*p* > 0.5).

#### 3.6.2. MDR vs. Non-MDR Pathogens

Patients with MDR infections required CRRT more frequently and had longer LOS, but survival outcomes did not differ significantly by sequencing strategy.

#### 3.6.3. APACHE II Severity Tertiles

**Figure 4:**
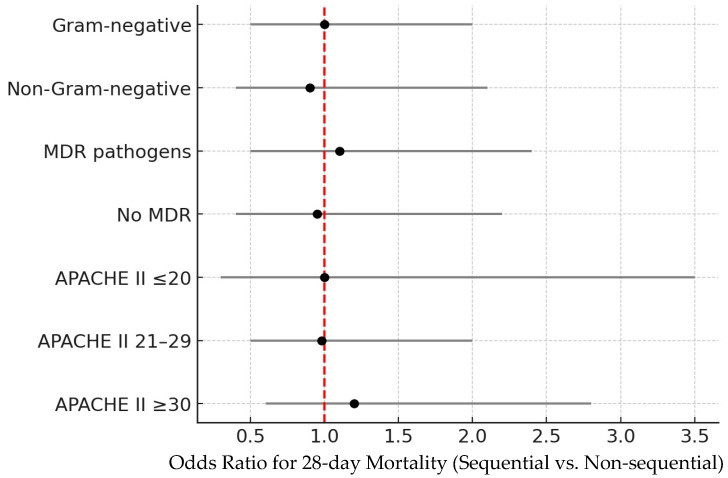
Subgroup analyses for 28-day mortality (forest plot) comparing sequential vs. non-sequential PMX-HP by infection type (Gram-negative vs. non-Gram-negative), MDR status, and APACHE II tertiles. All confidence intervals crossed unity. The red dashed vertical line represents the null value (odds ratio = 1.0), indicating no difference between sequential and non-sequential PMX-HP. The dots represent point estimates, and the horizontal lines indicate 95% confidence intervals.

Patients in the highest APACHE II tertile (≥30) experienced the highest mortality regardless of sequencing. No survival benefit from sequential therapy was observed in any tertile (Table 3, Figure 4).

### 3.7. Multivariate Predictors of Mortality

Multivariate logistic regression identified the following independent predictors of 28-day mortality (Table 4):

- Higher APACHE II score (adjusted OR 1.08, 95% CI 1.01–1.15, *p* = 0.02).
- Positive VIS change (T3–T1 > 0) (adjusted OR 1.17 per unit increase, 95% CI 1.05–1.32, *p* = 0.006).
- CRRT requirement within 28 days (adjusted OR 4.12, 95% CI 1.35–12.54, *p* = 0.01).

**Table 4:** Multivariate logistic regression for predictors of 28-day mortality.

Variable	Adjusted OR	95% CI	*p*-Value
APACHE II score (per point)	1.08	1.01–1.15	0.02 *
VIS change (T3–T1 > 0)	1.17	1.05–1.32	0.006 *
CRRT within 28 days	4.12	1.35–12.54	0.01 *
Sequential vs. non-sequential	0.94	0.36–2.41	0.89
Age (per year)	1.01	0.97–1.05	0.62
Male sex	1.12	0.45–2.81	0.81

* Statistically significant at *p* < 0.05. Abbreviations: OR, odds ratio; CI, confidence interval; VIS, vasoactive-inotropic score; CRRT, continuous renal replacement therapy.

Sequential vs. non-sequential PMX-HP was not associated with mortality (adjusted OR 0.94, 95% CI 0.36–2.41, *p* = 0.89).

## 4. Discussion

In this single-center cohort of critically ill patients with severe sepsis and septic shock, we found that sequential polymyxin B hemoperfusion (PMX-HP) did not significantly improve survival compared with non-sequential therapy. Mortality rates at 28 days, ICU discharge, and hospital discharge were similar between groups. Patients treated with sequential PMX-HP exhibited longer hospital stays and higher rates of CRRT use. Importantly, these findings likely reflect greater baseline illness severity and organ dysfunction in patients selected for repeated treatment, rather than a detrimental effect of sequential PMX-HP itself. Prognosis was primarily determined by underlying disease severity (APACHE II score), renal dysfunction requiring CRRT, and persistent hemodynamic instability reflected by vasoactive-inotropic score (VIS) dynamics, rather than the sequencing of PMX-HP sessions. In particular, sepsis-associated acute kidney injury and severe organ dysfunction are well-recognized drivers of adverse outcomes in septic shock [26], and contemporary Sepsis-3 criteria emphasize organ dysfunction as central to sepsis and septic shock definition and risk stratification [27]. In addition, timely initiation of evidence-based sepsis care has a major impact on mortality and may outweigh the incremental effect of adjunctive extracorporeal therapies in unselected populations [28].

Our findings align with the broader literature questioning the survival benefit of PMX-HP in unselected septic shock populations. Although our results are consistent with prior studies, they reinforce the robustness of these observations in a real-world Asian ICU setting and highlight the dominant influence of illness severity and organ dysfunction over treatment sequencing. The EUPHAS trial reported improved survival and early organ recovery with PMX-HP in abdominal septic shock [9], generating early enthusiasm. However, subsequent larger RCTs, including ABDOMIX and EUPHRATES, failed to replicate these benefits [10,11]. In particular, the EUPHRATES trial, which enrolled patients with elevated endotoxin activity, did not demonstrate a mortality advantage, although post hoc analysis suggested potential benefit in patients with intermediate endotoxin activity levels [11].

Meta-analyses have further underscored this controversy. Some systematic reviews, including those by Terayama et al. and Li et al., concluded that PMX-HP may reduce mortality in specific subgroups, such as patients with Gram-negative infections or early treatment initiation [12,13]. In contrast, others have found no consistent benefit across heterogeneous populations [14]. A recent Japanese registry study also emphasized that outcomes may depend more on patient selection and baseline severity than on treatment modality itself [15]. Our data reinforce these observations: sequencing did not affect mortality, and outcome predictors were dominated by severity markers and organ dysfunction.

The rationale for sequential PMX-HP lies in the assumption that endotoxin release is sustained or biphasic, requiring repeated adsorption for effective clearance [18]. Early registry data from Japan suggested that two sessions might yield better hemodynamic stabilization [19]. However, other observational studies—including ours—have not confirmed survival benefits with sequential therapy [20].

A potential explanation is immortal time bias: patients must survive long enough to receive a second session, which may inherently select for those with better prognosis [21]. Additionally, doubling the number of sessions increases exposure to procedure-related risks, including hypotension, thrombocytopenia, and circuit clotting [22]. Our results showed no interaction between treatment sequencing and VIS dynamics, indicating that hemodynamic recovery was independent of whether therapy was sequential or not. Taken together, the evidence suggests that the number and timing of PMX-HP sessions may be less critical than appropriate patient selection and early recognition of hemodynamic response. Clinically, persistent vasodilatory shock requiring escalation of vasoactive support may reflect extreme circulatory failure; adjunctive agents such as angiotensin II have been evaluated in this context [29].

Independent of treatment sequence, higher APACHE II scores, CRRT requirement, and positive VIS change were strong predictors of mortality in our study. This is consistent with prior work demonstrating that disease severity and organ dysfunction dominate outcomes in septic shock [1,2]. The role of VIS as a prognostic marker has gained attention in recent years. Persistent or rising VIS has been associated with poor outcomes in both septic shock and cardiac surgery populations [22,23,24]. Our findings add to this evidence, showing that VIS dynamics are more informative for prognosis than the number of PMX-HP sessions.

More broadly, contemporary randomized trials highlight that outcomes in septic shock are strongly influenced by core resuscitation and adjunctive supportive strategies, including intravenous fluid strategies [30], choice of resuscitation targets (e.g., peripheral perfusion-based approaches versus lactate-driven strategies) [31], corticosteroid regimens [32,33], and vitamin C–based interventions with mixed findings across studies [34,35,36]. These data support the concept that overall physiologic trajectory and response to timely supportive care may be more decisive than the sequencing of a single adjunctive extracorporeal therapy.

Renal dysfunction also emerged as a decisive factor: patients requiring CRRT had markedly higher mortality, reinforcing the importance of early renal support as both a clinical marker and a therapeutic target [26]. These results highlight that critical illness trajectory, rather than extracorporeal therapy per se, drives survival.

The applicability of PMX-HP may differ between Asia and Western countries due to epidemiological and healthcare system factors. In East Asia, Gram-negative bacteria such as *E. coli* and *K. pneumoniae* predominate in sepsis [15,16], providing stronger rationale for endotoxin adsorption. Japan has integrated PMX-HP into its national guidelines, supported by reimbursement and the use of endotoxin activity assay (EAA) to guide initiation [20].

In Taiwan, PMX-HP is reimbursed by the NHI for refractory septic shock but without biomarker-based criteria [21]. This leads to variability in indications, timing, and session sequencing across institutions. While this policy facilitates access, it may also dilute treatment efficacy by including patients less likely to benefit. Our results suggest that a more selective approach, potentially incorporating biomarkers or dynamic severity measures, may improve both clinical and economic outcomes.

Several limitations must be acknowledged. First, this was a single-center retrospective study with a modest sample size, which limits generalizability. Second, we lacked a contemporaneous non-PMX-HP control group; thus, causal inferences regarding PMX-HP efficacy cannot be made. Third, endotoxin activity assay (EAA) was not routinely measured, precluding stratification by endotoxin burden. Fourth, sequential therapy was clinician-determined rather than protocolized, introducing potential selection bias. Fifth, immortal time bias may have influenced sequential outcomes, as patients needed to survive long enough to receive multiple sessions. Finally, some data (e.g., ventilator-free days) were incomplete due to real-world documentation variability. Notably, no patient in the non-sequential group underwent three or more PMX-HP sessions, precluding further subgroup stratification based on treatment frequency. Accordingly, the findings of this study should be interpreted as hypothesis-generating rather than definitive, and larger prospective studies are required to further validate these observations.

Future research should address these limitations by conducting multicenter, prospective trials with standardized protocols for PMX-HP initiation, session sequencing, and integration with CRRT. Biomarker-guided strategies and better standardized case definitions may improve patient selection and comparability across studies [27]. Combining VIS dynamics with other real-time markers (e.g., lactate clearance, SOFA trajectory, and perfusion-based endpoints) could yield precision-based algorithms for extracorporeal therapy and shock management [31,37]. Given the well-described heterogeneity of sepsis and septic shock, individualized, score-based management strategies warrant further evaluation [37,38]. Cost-effectiveness analyses are also warranted, particularly in health systems with universal coverage. Large-scale registries across Asia could help refine evidence-based criteria for PMX-HP use, ensuring equitable and efficient allocation of critical care resources. In parallel, S-AKI epidemiology and outcome studies should inform risk stratification and endpoint selection [26], and survivorship-focused outcomes (recovery and post-sepsis trajectory) should be considered in future designs [39].

## Figures and Tables

**Table 1:** Baseline characteristics of patients receiving sequential vs. non-sequential PMX-HP.

Characteristics	Total (*n* = 64)	Sequential (*n* = 33)	Non-Sequential (*n* = 31)	*p*-Value
Age, years, mean ± SD	66.1 ± 12.3	65.4 ± 11.8	66.9 ± 12.9	0.72
Male sex, *n* (%)	43 (67.2)	23 (69.7)	20 (64.5)	0.65
Body weight, kg, mean ± SD	66.5 ± 15.2	67.1 ± 14.7	65.8 ± 15.9	0.81
APACHE II score, median (IQR)	26 (21–32)	27 (22–33)	25 (20–31)	0.44
Source of sepsis, *n* (%)				
- Pneumonia	19 (29.7)	10 (30.3)	9 (29.0)	0.91
- Intra-abdominal infection	12 (18.8)	7 (21.2)	5 (16.1)	0.62
- Urinary tract infection	11 (17.2)	6 (18.2)	5 (16.1)	0.82
- Others (SSTI, liver abscess, bacteremia)	22 (34.3)	10 (30.3)	12 (38.7)	0.48
MDR pathogens, *n* (%)	17 (26.6)	9 (27.3)	8 (25.8)	0.89

Abbreviations: SD, standard deviation; IQR, interquartile range; APACHE II, Acute Physiology and Chronic Health Evaluation II; MDR, multidrug-resistant; SSTI, skin and soft tissue infection. Percentages may not total 100% due to rounding.

**Table 2:** Clinical outcomes of patients by treatment group.

Outcomes	Total (*n* = 64)	Sequential (*n* = 33)	Non-Sequential (*n* = 31)	*p*-Value
28-day mortality, *n* (%)	30 (46.9)	15 (45.5)	15 (48.4)	0.82
ICU mortality, *n* (%)	34 (53.1)	17 (51.5)	17 (54.8)	0.77
Hospital mortality, *n* (%)	34 (53.1)	17 (51.5)	17 (54.8)	0.77
ICU LOS, days, median (IQR)	9.3 (4.4–21.1)	11.1 (5.2–22.4)	7.8 (3.5–18.7)	0.09
Hospital LOS, days, median (IQR)	20.5 (8.0–34.6)	24.8 (10.2–38.5)	14.2 (6.0–28.1)	0.03 *
CRRT use, *n* (%)	40 (62.5)	23 (69.7)	17 (54.8)	0.04 *
ECMO use, *n* (%)	3 (4.7)	2 (6.1)	1 (3.2)	0.63

* Statistically significant at *p* < 0.05. Abbreviations: ICU, intensive care unit; LOS, length of stay; CRRT, continuous renal replacement therapy; ECMO, extracorporeal membrane oxygenation.

**Table 3:** Subgroup outcomes by infection type, MDR status, and APACHE II tertiles.

Subgroup	Sequential Mortality (%)	Non-Sequential Mortality (%)	*p*-Value
Gram-negative infections	15/30 (50.0)	15/30 (50.0)	0.99
Non-Gram-negative infections	3/9 (33.3)	2/7 (28.6)	0.81
MDR pathogens present	5/9 (55.6)	5/8 (62.5)	0.73
No MDR pathogens	10/24 (41.7)	10/23 (43.5)	0.88
APACHE II ≤ 20	1/7 (14.3)	1/8 (12.5)	0.92
APACHE II 21–29	6/13 (46.2)	5/12 (41.7)	0.81
APACHE II ≥ 30	8/13 (61.5)	9/11 (81.8)	0.29

Abbreviations: MDR, multidrug-resistant; APACHE II, Acute Physiology and Chronic Health Evaluation II.

## Data Availability

The original contributions presented in this study are included in the article. Further inquiries can be directed to the corresponding author.
